# MicroRNA-27b Impairs Nrf2-Mediated Angiogenesis in the Progression of Diabetic Foot Ulcer

**DOI:** 10.3390/jcm12134551

**Published:** 2023-07-07

**Authors:** Shukla Sakshi, Ravichandran Jayasuriya, Rajappan Chandra Sathish Kumar, Dhamodharan Umapathy, Athira Gopinathan, Ramachandran Balamurugan, Kumar Ganesan, Kunka Mohanram Ramkumar

**Affiliations:** 1 Department of Biotechnology, School of Bioengineering, SRM Institute of Science and Technology, Kattankulathur 603 203, Tamil Nadu, India; sakshishukla848@gmail.com (S.S.); jayasuriya199@gmail.com (R.J.); usadhamodharan@gmail.com (D.U.); 2 Interdisciplinary Institute of Indian System and Medicine (IIISM), SRM Institute of Science and Technology, Kattankulathur 603 203, Tamil Nadu, India; ayursatish@gmail.com; 3 SRM Medical College Hospital and Research Centre, SRM Institute of Science and Technology, Kattankulathur 603 203, Tamil Nadu, India; drathirag@gmail.com (A.G.); balamurr@srmist.edu.in (R.B.); 4 School of Chinese Medicine, LKS Faculty of Medicine, University of Hong Kong, Hong Kong 999077, China; kumarg@hku.hk

**Keywords:** microRNA-27b, impaired angiogenesis, *Nrf2*, DFU, endothelial cells

## Abstract

Nuclear factor erythroid-2-related factor 2 (*Nrf2*) is a stress-activated transcription factor regulating antioxidant genes, and a deficiency thereof, slowing lymphangiogenesis, has been reported in diabetic foot ulcer (DFU). The mode of *Nrf2* regulation in DFU has been less explored. Emerging studies on miRNA-mediated target regulation show miRNA to be the leading player in the pathogenesis of the disease. In the present study, we demonstrated the role of miR-27b in regulating *Nrf2*-mediated angiogenesis in DFU. A lower expression of mRNA targets, such as *Nrf2*, *HO-1*, *SDF-1α*, and *VEGF*, was observed in tissue biopsied from chronic DFU subjects, which was in line with miR-27b, signifying a positive correlation with *Nrf2*. Similarly, we found significantly reduced expression of miR-27b and target mRNAs *Nrf2*, *HO-1*, *SDF-1α*, and *VEGF* in endothelial cells under a hyperglycemic microenvironment (HGM). To confirm the association of miR-27b on regulating *Nrf2*-mediated angiogenesis, we inhibited its expression through RNA interference-mediated knockdown and observed disturbances in angiogenic signaling with reduced endothelial cell migration. In addition, to explore the role of miR-27b and angiogenesis in the activation of *Nrf2*, we pretreated the endothelial cells with two well-known pharmacological compounds—pterostilbene and resveratrol. We observed that activation of *Nrf2* through these compounds ameliorates impaired angiogenesis on HGM-induced endothelial cells. This study suggests a positive role of miR-27b in regulating *Nrf2*, which seems to be decreased in DFU and improves on treatment with pterostilbene and resveratrol.

## 1. Introduction

Diabetic foot ulcer (DFU) is one of the major microvascular complications in patients with type 2 diabetes mellitus (T2DM) and has been a significant threat to public health for many decades [1]. DFU patients are reported to experience neuropathic pain of origin followed by prickling at the foot site and further extending to numbness of the foot, which may result in leg amputations if untreated [2]. It is estimated that about 40 to 60 million people with diabetes suffer the burden of chronic ulcers worldwide, which can end in lower-limb amputations and a significant reduction in life expectancy [3]. Poor glycemic control in diabetic patients paves the way for chronic non-healing wounds associated with persistent infection leading to biofilm formation and prolonged inflammation followed by insufficient angiogenesis [4].

Many studies have focused on the association between poor blood flow and impaired angiogenesis in DFU patients [5]. Lack of oxygen at the injured site results in hypoxia, which plays a vital role in activating the endothelial cells (ECs). Endothelial progenitor cells (EPCs) are homed by the secretion of angiogenic factors such as stromal derived factor-1α (SDF-1α) and vascular endothelial growth factor (VEGF) [5]. A chronic oxidative milieu experienced at the wound site retards the release of these proangiogenic markers, resulting in low EPC mobilization [5,6].

A recent report from our laboratory identified nuclear factor erythroid-2-related factor 2 (*Nrf2*) as a prime target that indulges in the transcription of antioxidant genes that counteract oxidative stress. We have also reported reduced levels of *Nrf2* in diabetic patients and its involvement in regulating angiogenesis in DFU patients [7]. Studies have proven the association of Nrf2 signaling in other pathological conditions, such as fatty liver disease [8], neurodegenerative diseases [9], and cancer [10,11]. Under normal conditions, the level of *Nrf2* is maintained by its endogenous inhibitor, Kelch-like ECH-associated protein 1 (*Keap1*), which ubiquitinates *Nrf2* upon its covalent binding. In a stress environment, the *Nrf2*–*Keap1* complex disassociates, thereby localizing *Nrf2* in the nucleus and promoting the transcription of antioxidant genes [12]. Recent literature from Yu et al. reviewed upstream protein kinases, such as PI3K, PKC, and ERK, that phosphorylate Nrf2 and promote antioxidant signaling in response to oxidative stress [13]. Apart from these traditional upstream kinases, a few studies have identified microRNAs (miRNAs/miRs) that act upstream to the target and modify its transcription [14,15].

miRNAs are a class of small non-coding transcripts that partly drive the epigenetic machinery and are intricate in gene regulation [16,17]. Interest in finding the role of miRNAs that participate in the signaling pathway of many diseases has been growing in recent years. Many studies have found miRNAs that participate in post-transcriptional gene regulation by binding with a target mRNA, enhancing mRNA degradation or translational suppression [18]. In our current study, we aimed to study the role of *Nrf2* regulating miRNAs, which may activate Nrf2 and ameliorate the worsened angiogenesis in DFU subjects. A few miRNAs, including miR-193b, miR-29b, miR-181c, miR-617, miR-32, miR-592, miR-220c, and miR-27b, have already been reported to regulate *Nrf2* in various diseases [19]. Hence, in this study, we first aimed to screen potent miRNAs through a bioinformatics approach and study their role in regulating *Nrf2*-mediated angiogenesis in DFU subjects. We also checked the miRNA-mediated *Nrf2* regulation of selected miRNAs through well-known pharmacological activators of *Nrf2*. Resveratrol (RES) is a polyphenol reported to have many beneficial activities, e.g., antioxidant, anti-inflammatory, antiaging, etc. [20]. Studies have reported that RES accelerated wound healing in H_2_O_2_-induced endothelial cells through increasing Nrf2-mediated angiogenesis by combating oxidative stress [21]. Another study by Huang et al. found healing of diabetic wounds through RES-mediated enhanced migration and proliferation of hyperglycemia-induced human umbilical vein endothelial cells (HUVECs) in vitro [22]. Yet another pharmacological compound, pterostilbene (PTS) is a stilbenoid chemically related to RES and has been found to have more medicinal properties, e.g., anti-inflammatory, antidiabetic, antioxidant, etc. [23]. A recent report has proved PTS mediated acceleration of wound healing by modulating epigenetic changes in hematopoietic stem cells in diabetic rats [24]. Further, topical administration of PTS resulted in improved wound healing through activation of *HIF-1α* signaling in diabetes-induced rats [25]. In this context, one of our recent reports demonstrated the *Nrf2*-activation potential of PTS in heme-induced macrophage dysregulation under hyperglycemic microenvironment [26].

## 2. Materials and Methods

### 2.1. Identification of Target miRNA through In Silico Approach

To identify target miRNAs, a systematic literature review was performed using Science Direct, Web of Science, PubMed, Google, Embase, and the Scientific Information Database (SID). The survey included the keywords miRNA, microRNA, miRNA and angiogenesis, miRNA and DFU, miRNA and diabetes, miRNA and *Nrf2*, *Nrf2* regulating miRNA, miRNA regulating *Nrf2*, *Nrf2* and angiogenic miRNAs. The collected data were then uploaded to the MirMap bioinformatics tool (https://mirmap.ezlab.org/ (accessed on 26 August 2022)) to predict target miRNAs with our gene of interest, *Nrf2*. MirMap computed and assigned individual scores, from which we selected miR-27b.

### 2.2. Enrollment of the Study Subjects

A total of 22 DFU tissue samples were collected from SRM Medical Hospital, Kattankulathur with institutional ethical committee approval (1901/IEC/2020). The study was subcategorized into acute DFU (grade 1; *n* = 10) and chronic DFU (≥grade 2; *n* = 12) based on the severity of the ulcers that were examined by a podiatrist at the time of debridement. As per the IWGDF/IDSA system of DFU classification, wounds without any clinical manifestations and in an uninfected state were recognized as grade 1 and represented acute DFU. Chronic foot ulcer cases are seen with varied clinical manifestations and infection rates from mild to severe based on the severity of grades. Patients with grade 2 have limited or mild infection in the skin or superficial subcutaneous tissue. A moderate infection as seen in grade 3 cases, extends to muscle, tendon, joint or bone. Grade 4, categorized as severe infection, is associated with systemic toxicity or metabolic instability. Hence, grades 2–4 are categorized as infected or chronic foot ulcers. The patients had the study briefly explained to them in English and their native language before obtainment of informed consent. The study included subjects with at least 10 years of diabetes with the progressive development of ulcers. Patients who underwent simultaneous treatment with anti-inflammatory drugs were excluded from this study. Patients were injected with 1% lidocaine to induce local anesthesia, after which a full-thickness wound (approximately 100 mg) was taken using a trephine/punch biopsy. A punch biopsy of 6 mm diameter and 2–3 mm depth was used to collect tissue from the participants. The collected samples were washed several times with PBS to remove the blood streaks, transferred to an RNA-stabilizing agent (Qiagen, Germantown, MD, USA), and stored at −80 °C until further use.

### 2.3. Cell Culturing Conditions and Treatments

The human endothelial cell line EA.hy926 was cultured in DMEM supplemented with 10% FBS and maintained in a humidified incubator with 5% CO_2_. The cells were maintained in DMEM with 1% reduced serum for at least 2 h before treatment. Then, cells were exposed to a hyperglycemic microenvironment (HGM) by adding 33.3 mM extra glucose [27] and 10 ng/mL each of TNF-α and IFN-γ for 24 h. The cells were treated with Nrf2 activators such as PTS and RES at 5 and 10 µM concentrations for 8 h [28,29].

### 2.4. Isolation of miRNA and mRNA

The tissue samples frozen in RNA-stabilizing agent were retrieved and washed twice with PBS. Approximately 50 mg of tissue was weighed and transferred to a sterile RNase-free Eppendorf tube with 500 µL of QIAzol (Qiagen, Germantown, MD, USA). The samples were chopped with scissors and minced using a handheld homogenizer maintained on ice. The homogenate was mixed thoroughly by vortexing for about 30 min. In parallel, the cell pellets were homogenized in 300 µL of QIAzol with occasional vortexing for almost 30 min. After this, 150–200 µL of chloroform was added to the tissue, and the cell homogenate was incubated on ice with gentle mixing. The mixtures were then centrifuged at 12,000 rpm for 15 min at 4 °C. The clear supernatant was transferred to a new sterile Eppendorf tube and mixed with an equal volume of 70% ethanol. The contents were then transferred to a mini spin column provided with an miRNeasy mini kit (Qiagen, Germantown, MD, USA). This kit allows the binding of larger RNA, including mRNA, and the small RNAs are collected as flow-through. The contents were centrifuged at 10,000 rpm for a minute at room temperature. The flow-through was collected in a new sterile Eppendorf tube and the spin column was stored at 4 °C for a short period of time. This flow-through contained small RNA, including miRNA, and hence it was mixed with a quarter volume of 100% ethanol (Hayman, Witham, UK) and transferred to the mini spin column of another kit (RNeasy MinElute Cleanup Kit, Qiagen, Germantown, MD, USA). This cleanup kit helps to isolate small RNA-enriched fractions that contain miRNA. The spin column was centrifuged at 10,000 rpm for a minute and the flow-through was discarded. A series of washes were performed on the spin column, as recommended by the manufacturer. The miRNA-containing fraction was eluted from the column using nuclease-free water (BioBasic, Markham, Canada) and stored at −80 °C till further use. Next, the spin column stored at 4 °C was taken out and washed with buffers provided with the kit as per the manufacturer’s recommendation. Finally, the large RNA fraction containing mRNA was eluted in a desired volume of nuclease-free water and stored at −80 °C till further use.

### 2.5. cDNA Conversion and Real-Time PCR (RT-PCR)

RNA concentration was quantified using a NanoDrop™ 2000c Spectrophotometer (Thermo Fisher Scientific, Waltham, MA, USA), and the samples with purity 2.0 were further converted to cDNA. Each RNA (1 µg) was used for further conversion. A miScript II kit (Qiagen, Germantown, MD, USA) was used to convert small RNA-enriched fractions to miRNA-cDNA. The study primarily focused on mature miRNAs, and hence we used Hi spec buffer from the provided kit (as per the manufacturer’s instruction). The Iscript cDNA synthesis kit (Bio-Rad, Hercules, CA, USA) was used to synthesize mRNA-cDNA from larger RNA-enriched fractions.

The expression of miRNA and mRNA of interest was determined using gene-specific primers (Table S1) in a Quantstudio 5 qPCR instrument (Applied Biosystems, Thermo Fisher Scientific, Waltham, MA, USA). A miScript SYBR Green PCR Kit (Qiagen, Germantown, MD, USA) was used to determine the expression of miR-27b and U6 as an internal control. For mRNA-cDNA, GoTaq qPCR master mix (Promega, USA) was used along with gene-specific primers and GAPDH as an internal control.

### 2.6. Silencing of miR-27b in Endothelial Cells Using Antisense Oligonucleotides

Antisense oligonucleotides (ASOs) targeting miR-27b (Si-miR27b) and a non-target scrabble control (Sc) were synthesized with the sequence Si-miR-27b: GCAGAACTTAGCCACTGTGAA and Sc: ACGTCTATACGCCCA. Both ASOs were transiently transfected into 60% confluence endothelial cells using Lipofectamine 3000 (Invitrogen, Thermo Fisher Scientific, Waltham, MA, USA). After 4 h of transfection, the Lipofectamine complex was replaced with DMEM with 10% FBS. The cells were then harvested for RNA isolation.

### 2.7. Scratch Assay

The cells were transiently transfected, as mentioned in Section 2.6. Once the cells became 100% confluent, the monolayer was scratched using a 10 µL sterile tip. The migration of cells was examined periodically after scratching, and photographs were recorded at 0 h, 8 h, and 12 h using an inverted microscope.

### 2.8. Statistical Analysis

Statistical significance between any two groups was analyzed using Student’s *t*-test, whereas significance more than two comparable groups, was determined using one-way ANOVA. All statistical analyses in bar graphs were performed using GraphPad Prism software (v. 8.0), where *p* < 0.05 was considered statistically significant. The expression of miR-27b was correlated with *Nrf2* using Spearman’s rank correlation using SPSS software (v. 20.0). All bar graphs were plotted using GraphPad Prism (v. 8.0) and the correlation plot using SPSS software (v. 20.0). The images of the scratch assay were quantified using ImageJ software (v. 1.53t).

## 3. Results

### 3.1. Clinical Parameters of the Study Subjects

**Figure 1:**
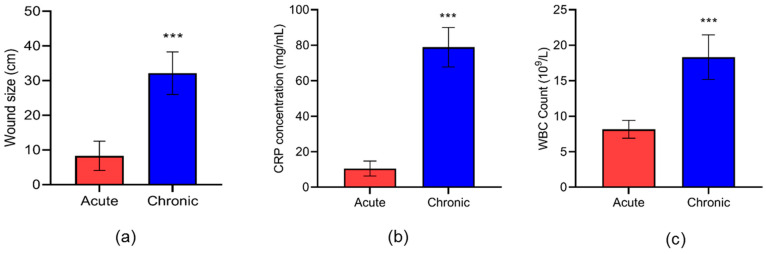
Comparison of wound size (**a**), CRP (**b**) and WBC count (**c**) in patients with acute and chronic DFU. Data are presented as mean ± S.D. *** *p* < 0.001. *n* = 22 (acute = 10; chronic = 12).

Table 1 presents the biochemical parameters of the study subjects. HbA1c and LDL-c were found to be significantly elevated in chronic compared to acute DFU subjects. Wound size was observed to be significantly larger in chronic than acute DFU patients (Figure 1a). We also observed an increased level of CRP and WBC in chronic compared to acute DFU patients, with a significance of *p* < 0.001 (Figure 1b,c).

### 3.2. Gene Expression Analysis of Nrf2 and Its Downstream Targets among the Study Subjects

**Figure 2:**
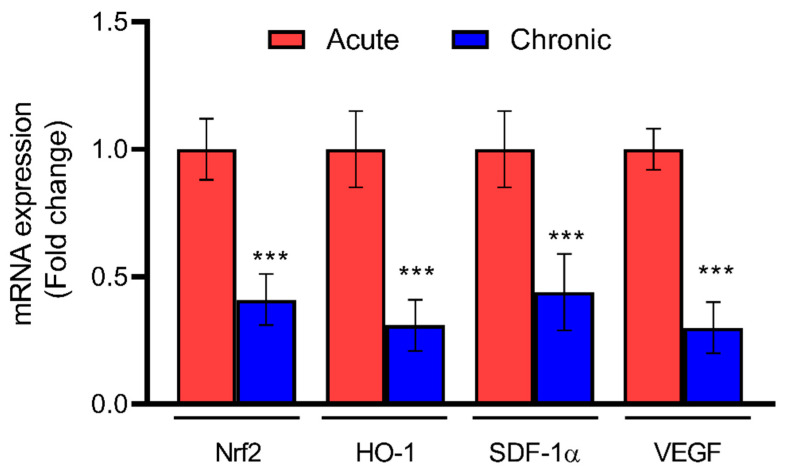
Relative mRNA expression of tissue-specific angiogenic markers among patients with acute and chronic DFU assessed by qPCR. Data are presented as mean ± S.D. *** *p* < 0.001. *n* = 22 (acute = 10; chronic = 12).

The gene expressions of *Nrf2*, *HO-1*, and angiogenic markers such as *SDF-1α* and *VEGF* were assessed using qPCR and are depicted in Figure 2. A significant decrease in the expression of *Nrf2* (*p* < 0.001) and its downstream target *HO-1* (*p* < 0.001) was observed: 2.5- and 3.5-fold, respectively. Similarly, on analyzing the gene expression of angiogenic markers, a 2.5-fold and 3-fold decrease was observed for *SDF-1α* and *VEGF*, respectively, with a significance of *p* < 0.001.

### 3.3. Gene Expression Analysis of miR-27b and Its Correlation with Nrf2 among the Study Subjects

**Figure 3:**
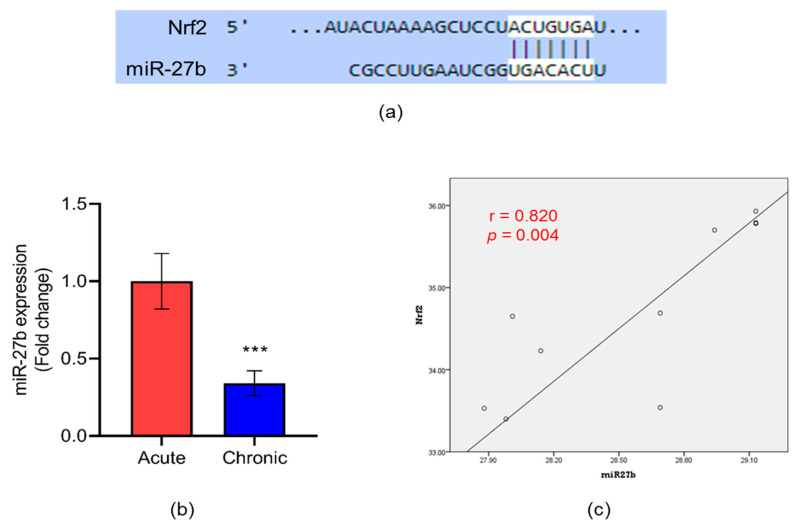
(**a**) Targeted binding site of miR-27b with *Nrf2* predicted by target scan. (**b**) Relative expression of tissue-specific miR-27b in patients with acute and chronic DFU assessed by qPCR. Data are presented as mean ± S.D. [*** *p* < 0.001]. (**c**) Spearman’s correlation of tissue-specific miR-27b with *Nrf2* among the study subjects was determined using SPSS software, version 20. *p* and r were calculated at 95% confidence intervals. *n* = 22 (acute = 10; chronic = 12).

To identify putative targets of *Nrf2*, we carried out an extensive literature survey, as mentioned in Section 2.1. The selected miRNAs were further analyzed using miRmap to confirm the association with *Nrf2*. Based on the score (88.31) and type of binding site (7 mer), miR-27b was selected for this study and the target binding site was predicted using a target scan (Figure 3a). As shown in Figure 3b, the expression of miR-27b was significantly lower (3.5-fold, *p* < 0.001) in chronic than acute DFU subjects. We found a positive correlation between miR-27b and *Nrf2* with a correlation coefficient (r) and significance (*p*) of 0.820 and 0.004, respectively, among chronic DFU subjects (Figure 3c).

### 3.4. qPCR Analysis of miR-27b and Angiogenic Markers on the Hyperglycemic Microenvironment in Endothelial Cells

**Figure 4:**
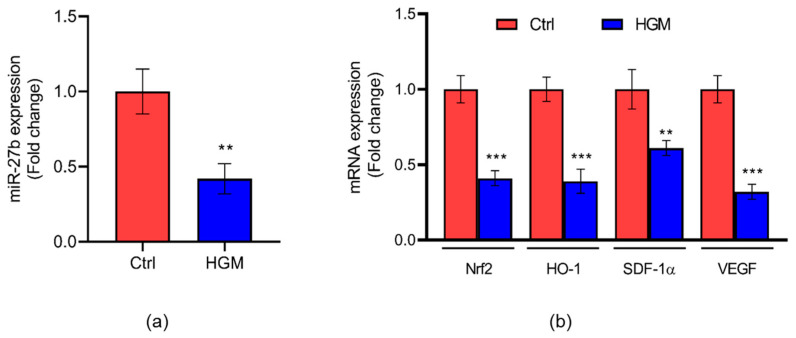
(**a**) Expression of miR-27b on hyperglycemic microenvironment-induced endothelial cells using qPCR. Data are presented as mean ± S.D. [** *p* < 0.01]. (**b**) Expression of angiogenic markers on HGM-induced endothelial cells assessed using qPCR. Data are presented as mean ± S.D. ** *p* < 0.01; *** *p* < 0.001. Control and hyperglycemic microenvironment are represented as Ctrl and HGM in this figure. *n* = 3.

The expression of miR-27b (Figure 4a) was significantly reduced (2.5-fold; *p* < 0.01) at 24 h of exposure to HGM in endothelial cells. The mRNA expression of *Nrf2* (2.5-fold; *p* < 0.001), *HO-1* (2.5-fold; *p* < 0.01), *SDF-1α* (1.5-fold; *p* < 0.05) and *VEGF* (5-fold; *p* < 0.001) was also found to be significantly decreased in HGM-induced endothelial cells compared to control (Figure 4b).

### 3.5. Effect of Silencing miR-27b on the Regulation of Angiogenesis in Endothelial Cells

**Figure 5:**
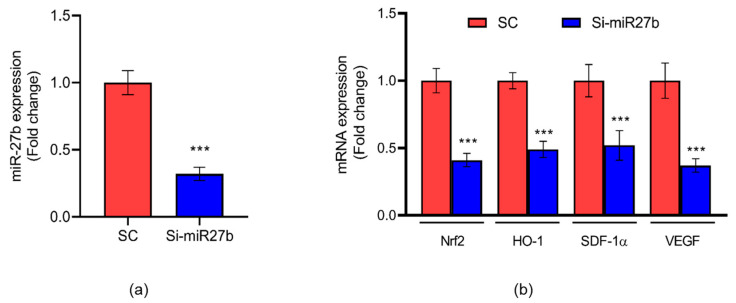
(**a**) Inhibition of miR-27b expression in endothelial cells using an antisense oligonucleotide (ASO). Data are presented as mean ± S.D. *** *p* < 0.001. (**b**) Effect of inhibiting miR-27b on the regulation of angiogenesis in endothelial cells. Data are presented as mean ± S.D. *** *p* < 0.001. Endothelial cells transfected with ASO targeting miR-27b are represented as Si-miR27b and Sc is represented as Sc. *n* = 3.

**Figure 6:**
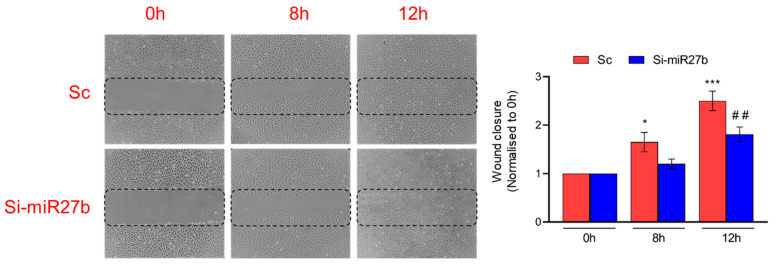
Effect of endothelial cell migration on inhibiting miR-27b assessed by scratch assay. Data are presented as mean ± S.D. * *p* < 0.05; ## *p* < 0.01, *** *p* < 0.001. Endothelial cells transfected with ASO targeting miR-27b are represented as Si-miR27b and scramble control is represented as Sc. * Compared to 0 h of Sc; # compared to 0 h of Si-miR27b. *n* = 2.

To study the role of miR-27b in the regulation of angiogenesis, miR-27b was silenced in endothelial cells. As shown in Figure 5a, the expression of miR-27b was significantly reduced (2.5-fold; *p* < 0.05) in Si-miR27b-transfected cells compared to those transfected with scrambled control (Sc). Similarly, we observed a decreased expression of *Nrf2* (2.5-fold; *p* < 0.01), *HO-1* (1.5-fold; *p* < 0.05), *SDF-1α* (1.5-fold, *p* < 0.05) and *VEGF* (2.5-fold; *p* < 0.001) (Figure 5b). We studied cell migration by scratch assay on the cells transfected with Sc and Si-miR-27b. Compared to cells transfected with Sc, Si-miR27b showed poor migration and closure of the wound. In the cells transfected with Sc, we observed cell migration at 8 h, which was completed at 12 h (*p* < 0.001). In the cells transfected with Si-miR-27b, we observed a slow migration of cells at 8 h, with no complete migration even at 12 h (Figure 6).

### 3.6. Effect of PTS and RES on miR-27b-Regulated Angiogenesis in Hyperglycemic Microenvironment-Induced Endothelial Cells

**Figure 7:**
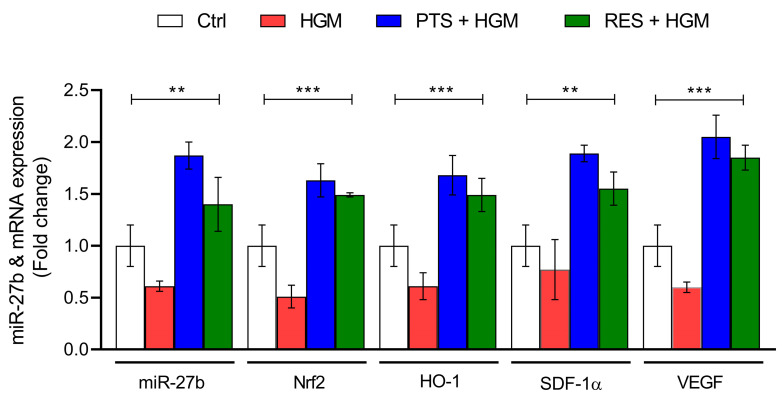
Effect of PTS and RES on miR-27b-regulated angiogenesis in endothelial cells under a hyperglycemic microenvironment. Data are presented as mean ± S.D. ** *p* < 0.01; *** *p* < 0.001. Pterostilbene and resveratrol are abbreviated as PTS and RES, respectively. *n* = 3.

Foreseeing miR-27b as an upstream target of Nrf2, we aimed to understand the changes in the expression pattern of miR-27b upon treatment with two well-known Nrf2 activators—pterostilbene and resveratrol. The effect of these Nrf2 activators on the expression of miR-27b, *Nrf2*, *HO-1*, *SDF-1α*, and *VEGF* was assessed under normoglycemic conditions. On analyzing the same in HGM-induced endothelial cells pretreated with PTS and RES, we found significantly increased expression of miR-27b and other angiogenic mRNAs compared to cells exposed to a hyperglycemic microenvironment only (Figure 7).

## 4. Discussion

DFU is one of the severe complications of T2DM, impedes common angiogenic mechanisms, and is associated with chronic oxidative stress at the injured site, thereby causing poor closure of wounds [30]. Research on the molecular mechanisms involved in diagnosing these complex wounds is still lacking. Angiogenesis is regulated by several mechanisms, among which the most extensively explored are miRNAs [31]. miRNAs have gained much interest recently, as they have been identified as biomarkers in many diseases. This potential epigenetic tool alters the gene expression pattern, which affects the pathogenesis or severity of diseases [32]. In this way, we identified dysregulated expression of miR-27b in DFU patients and attempted to understand its molecular role in regulating angiogenesis under HGM in vitro.

Angiogenesis plays a significant role in wound healing, wherein the lapse of proangiogenic growth factors has been established to be linked to the severity of DFU subjects [31]. Angiogenic growth factors, especially SDF-1α and VEGF, recruit and nourish the EPCs and the proper accomplishment of the proliferative phase [33]. Several reports have highlighted lessened angiogenic growth factors in DFU subjects [34,35,36]. Consistent with the available reports, our data from the current study also showed reduced expression of angiogenic factors such as *SDF-1α* and *VEGF* in the tissue of chronic compared to acute DFU subjects. Several studies support these findings, and recently our research group reported an increased expression of angiogenic markers in DFU patients who underwent hyperbaric oxygen (HBO) therapy [37].

When VEGF is well established as the master regulator of angiogenesis, Nrf2 is also thought to be a multifaceted regulator of antioxidant responses [38]. This transcription factor primarily activates a battery of antioxidant genes, including heme oxygenase 1 (HO-1), catalase (CAT), superoxide dismutase (SOD), NAD(P)H dehydrogenase (quinone) 1 (NQO1) and glutathione S-transferase (GST), in response to stress mediators under various stimuli [39]. Studies have demonstrated reduced angiogenesis upon the knockdown of *Nrf2* in vitro [35,40]. Li et al. reported the inhibition of Nrf2-ARE and the HIF-1α/VEGF signaling pathway upon knockdown of *Nrf2* [41]. Huang et al. studied the regulation of angiogenesis through the Nrf2/HO-1 axis, targeting *VEGF* [42]. In this way, we observed reduced expression of *Nrf2* and its downstream target *HO-1* in the tissue of chronic compared to acute DFU subjects. Our previous report suggests *Nrf2* as upstream of *VEGF*, confirmed using CRISPR knockout [43]. We also established a positive correlation between *Nrf2* and *VEGF* in chronic DFU subjects, supporting our current data [43]. Thus, lowered expression of *Nrf2*, *HO-1*, *SDF-1α* and *VEGF* in chronic DFU subjects suggests a hindrance in angiogenic signaling, which impedes the proper closure of wounds. These disturbances in angiogenic signaling are associated with a few upstream factors, one of the more frequent being kinases. For example, Jain et al. identified GSK-3β as inhibiting Nrf2 activation and nuclear transport through Fyn kinase in the HepG2 cell line [44]. Another study by Tan et al. on one another upstream kinase, ERK, showed suppression of *Nrf2*, and thereby oxidative stress-induced insulin resistance in cardiomyocytes [45].

In parallel, such molecular alterations in DFU are also demonstrated to be regulated by epigenetic mediators in addition to transcriptional and post-transcriptional events [46]. Epigenetic alterations include DNA methylation by DNA methyltransferases (DNMTs), histone modification by histone deacetylases (HDACs) and regulation of gene expression by non-coding RNAs (ncRNAs). For example, Hafez et al. identified the significant role of sirtuin-1 in DFU patients [47]. We have profiled multiple HDACs and correlated them with the expression of *Nrf2* in patients with T2DM and DFU [48]. On the other hand, many studies have identified the potential of miRNA in the pathogenesis and development of DFU [49,50,51,52]. miRNAs, which are classified under small ncRNAs, are one of the major epigenetic tools to have been widely explored and used as therapeutic targets [53,54,55].

A previous report from our research group found increased expression of miR23c, which negatively regulated *SDF-1α* in circulation and tissue biopsies of DFU subjects [56]. A few other reports have identified angiogenesis-regulating miRNAs such as miR-10a, miR-17–92, miR-126, miR-130a, miR-132, miR-210, miR-218, miR-221/222, miR-296, and miR-320 in different physiological and pathological conditions [57]. Hence, in this study, we examined miRNAs that act upstream of *Nrf2* and regulate angiogenic signaling under various stimuli. To this end, we performed a literature search of scholarly databases and used the bioinformatics approach to find miR-27b as a putative target of Nrf2. We then observed the expression of miR-27b in chronic DFU subjects which was lessened and in line with the expression of other angiogenic markers. Moreover, Spearman’s correlation analysis revealed a positive correlation of miR-27b with Nrf2, and thus these findings further evidenced the crucial role of miR-27b in promoting angiogenesis in DFU patients.

A systemic pathological condition observed in almost all DFU patients is the dysregulation of the endothelium, implying an imbalance between vasodilation and vasoconstriction factors produced by the endothelial cells [5]. Endothelial cell dysfunction defines an impairment in the function of endothelial cells, disturbances in proliferation and forming of the capillary network, and migration, diapedesis, and tube formation properties [58]. A recent report identified the regulation of miR-27b through FOXO1 as a target to mitigate mitochondrial oxidative stress and inflammation in TNF-α-induced HUVECs [59]. Inhibition of miR-27b was demonstrated to alleviate brain injury in rats through Nrf2-ARE signaling [60]. The relationship between miR-27b, along with miR-130a and miR-210, and regulation of oxidative stress was established by Signorelli et al. in patients with peripheral artery disease [61]. Urbich et al. has reported that miR-27a/b promotes angiogenesis by targeting an angiogenesis inhibitor, semaphorin 6A, in embryonic zebrafish. They also showed endothelial cell sprouting upon overexpression of miR-27a/b in HUVECs [62]. In our present study, the human endothelial cells were exposed to high glucose (33.3 mM) and a cytokine cocktail for 24 h to mimic a hyperglycemic microenvironment. We observed reduced expression of miR-27b along with Nrf2, HO-1, and angiogenic markers such as SDF-1α and VEGF. These findings further validated our observations in tissue biopsies of DFU patients. Tube formation ability is significantly reduced in the diabetic environment [63]. Similarly to our *in vitro* results, Ganesh et al. witnessed lessened protein expression of Nrf2 and HO-1 in macrophages under HGM in vitro, where similar experimental conditions were employed to mimic diabetic conditions [64]. Furthermore, Falco et al. and Kim et al. observed the same patterns of SDF-1α and VEGF in endothelial cells under HGM contributing to poor angiogenesis in vitro [65,66]. In contrast to our findings, Rong et al. reported that miR-27b suppressed the proliferation and migration of endothelial cells in Kawasaki disease [67].

To confirm the role of miR-27b in endothelial cells in angiogenesis, we silenced the expression of miR-27b using ASO-mediated RNA interference (RNAi). Our data revealed significantly reduced expression of *Nrf2, HO-1, SDF-1α* and *VEGF* in miR-27b-silenced endothelial cells compared to the one transfected with scrambled control. We developed the miR-27b-silenced cells to examine cell migration and proliferation. We observed slower migration in miR-27b-silenced cells than the one transfected with scrambled control. Emphasizing the importance of cell proliferation and migration, these data evidenced the direct role of miR-27b as upstream of *Nrf2* in regulating cell proliferation and angiogenesis. Extensively studied is miRNA-mediated suppression of the target, where an increase in miRNA decreases the target mRNA, establishing a negative-feedback loop. Apart from direct regulation of Nrf2, there are miRNAs that indirectly regulate Nrf2 through suppressing its repressor complexes [19]. For instance, Eades et al. demonstrated an indirect relationship between miR-200a and *Nrf2* through suppressing its repressor *Keap1* in breast cancer cells, suggesting a positive-feedback mechanism between miR-200a and *Nrf2* [19,68]. Similarly, our study also established a positive correlation between *Nrf2* and miR-27b, where miR-27b can indirectly target *Nrf2* through *Keap1*. However, further studies are needed to check the association of miR-27b with *Keap1* degradation and *Nrf2* activation.

Impaired angiogenesis or insufficient angiogenic markers are plausible factors causing DFU that are attributed to delayed healing of ulcers, which was also identified in our current study. Targeting the molecular pathways in angiogenesis is an upcoming novel and effective approach to overcoming life-threatening angiogenesis-dependent diseases, including cancer, diabetes, or other age-related diseases. The most common way to target specific genes is through sequence-based antisense oligonucleotide pairing, suppressing basal expression [69]. On the other hand, pharmacological modulation of targets by small molecules has broad potential to enable the biological function of genes and to develop a novel therapeutic strategy [70]. Many researchers are working on identifying and designing small molecules that bind with RNA, known as small-molecule interaction with RNAs (SMIRNAs) [71]. Hence, in our study, we attempted to observe the alteration in the expression pattern of miR-27b when exposed to two well-known Nrf2 activators (small molecules)—PTS and RES. With an increase in the expression of basal *Nrf2*, we observed increased miR-27b expression under normoglycemic conditions. To assess the expression profile in miR-27b-mediated Nrf2 signaling in angiogenesis, the endothelial cells were pretreated with PTS and RES, after which they were exposed to an HGM. Interestingly, we observed an ameliorative effect in the expressions of miR-27b, *Nrf2*, *HO-1*, *SDF-1α*, and *VEGF* when treated with either of these small molecules. Both the pharmacological compounds were found to activate *Nrf2* by increasing the expression of miR-27b. Topical administration of PTS was recently found to improve healing of wounds in diabetic rats through HIF-1α, which increased the expression of another angiogenic marker, VEGF [25]. On the other hand, RES has been reported to accelerate wound healing in H_2_O_2_-induced endothelial cells through increasing Nrf2-mediated angiogenesis by combating oxidative stress [21]. Hence, it could be hypothesized that PTS or RES activates *Nrf2* through miR-27b, which further increases the expression of angiogenic markers and improves healing of wounds. However, to elucidate the effect of these pharmacological compounds on miR-27b regulation, further studies are needed by inhibiting miR-27b and exposing them to these compounds. The activation of Nrf2 through Keap1 disassociation and translocation of free Nrf2 into the nucleus or activation through upstream kinases are well studied. Although the current study has demonstrated the transcription levels of *Nrf2* on inhibiting miR-27b through ASO, it is limited in elucidating the phosphorylation status of Nrf2 after inhibiting miR-27b.

## 5. Conclusions

In conclusion, this study found miR-27b to be a target for angiogenesis, which was decreased in chronic DFU patients. Inhibition of miR-27b reduced angiogenesis and cell migration in vitro. The *Nrf2* activators PTS and RES improved angiogenesis in endothelial cells mediated through miR-27b under HGM in vitro.

## Figures and Tables

**Table 1:** Clinical and biochemical parameters of the study subjects.

Clinical Parameters (*n* = 22)	Acute DFU (*n* = 10)	Chronic DFU (*n* = 12)
Gender (M/F)	3/7	7/5
Age (Years)	49.0 ± 4.9	51.2 ± 2.6
Body mass index (Kg/m^2^)	28.1 ± 1.6	29.1 ± 3.5
Systolic blood pressure (mm Hg)	135.7 ± 2.1	138.0 ± 4.6
Diastolic blood pressure (mm Hg)	85.4 ± 2.9	89.0 ± 4.1
Fasting plasma glucose (mg/dL)	191.7 ± 8.4	212.9 ± 16.4
Postprandial plasma glucose (mg/dL)	243.4 ± 9.6	279.0 ± 21.7
Glycated hemoglobin (%)	8.2 ± 1.6	10.2 ± 1.3 *
Total serum cholesterol (mg/dL)	182 ± 5.6	188.0 ± 6.8
HDL-cholesterol (mg/dL)	45.4 ± 8.1	40.0 ± 5.0
LDL-cholesterol (mg/dL)	93.4 ± 9.8	119.0 ± 19.8 *
Urea (mg/dL)	31 ± 3.5	35.8 ± 2.8
Creatinine (mg/dL)	1.0 ± 0.1	1.0 ± 0.3

All data are presented as mean ± S.D [* *p* < 0.05]. *p* values were compared between acute and chronic DFU subjects.

## Data Availability

Data available on request.

## Supplementary Materials

The following supporting information can be downloaded at https://www.mdpi.com/article/10.3390/jcm12134551/s1. Table S1. List of primers used in this study.
