# Supplementary material for: MicroRNA-27b Impairs Nrf2-Mediated Angiogenesis in the Progression of Diabetic Foot Ulcer
Source: J Clin Med. 2023 Jul 7;12(13):4551. doi: 10.3390/jcm12134551 (PMC10342788; doi:10.3390/jcm12134551)
Supplement: Supplementary file 1 [file jcm-12-04551-s001.zip › jcm-2446768-supplementary.pdf]

Supplementary Table

Table S1. List of primers used in this study.

Target name	Primer sequence	
	Forward	Reverse
**miR-27b**	TTCACAGTGGCTAAGTTCTGC	
**U6**	CGCAAGGATGACACGCAAATTC	
**Nrf2**	GACAATGAGGTTTCTTCGGCT	CGTCTAAATCAACAGGGGCTA
**HO-1**	GGGAATTCTCTTGGCTGGCT	AACTGAGGATGCTGAAGGGC
**SDF-1α**	CGCACTTTCACTCTCCGTCA	AGCACGACCACGACCTTG
**VEGF**	CTACCTCCACCATGCCAAGT	GCAGTAGCTGCGCTGATAGA
**GAPDH**	AAGAAGGTGGTGAAGCAGGC	GTCAAAGGTGGAGGAGTGGG
